# Presymptomatic and early pathological features of *MAPT*-associated frontotemporal lobar degeneration

**DOI:** 10.1186/s40478-023-01588-9

**Published:** 2023-08-02

**Authors:** Lucia AA Giannini, Merel O Mol, Ana Rajicic, Renee van Buuren, Lana Sarkar, Sanaz Arezoumandan, Daniel T Ohm, David J Irwin, Annemieke JM Rozemuller, Netherlands Brain Bank, John C van Swieten, Harro Seelaar

**Affiliations:** 1 Department of Neurology and Alzheimer Center Erasmus MC, Erasmus University Medical Center, Rotterdam, 3015 GD The Netherlands; 2 Digital Neuropathology Laboratory, Department of Neurology, Perelman School of Medicine, University of Pennsylvania, Philadelphia, PA 19104 USA; 3 Penn Frontotemporal Degeneration Center, Department of Neurology, Perelman School of Medicine, University of Pennsylvania, Philadelphia, PA 19104 USA; 4 Department of Pathology, Amsterdam Neuroscience, Amsterdam University Medical Center, location VUmc, Amsterdam, 1081 HZ The Netherlands; 5 Netherlands Institute for Neuroscience, Amsterdam, 1105 BA The Netherlands

**Keywords:** Frontotemporal lobar degeneration, *MAPT* gene, Early pathology, Neuronal degeneration, Tau burden

## Abstract

Early pathological features of frontotemporal lobar degeneration (FTLD) due to *MAPT* pathogenic variants (FTLD-MAPT) are understudied, since early-stage tissue is rarely available. Here, we report unique pathological data from three presymptomatic/early-stage *MAPT* variant carriers (FTLD Clinical Dementia Rating [FTLD-CDR] = 0–1). We examined neuronal degeneration semi-quantitatively and digitally quantified tau burden in 18 grey matter (9 cortical, 9 subcortical) and 13 white matter (9 cortical, 4 subcortical) regions. We compared presymptomatic/early-stage pathology to an intermediate/end-stage cohort (FTLD-CDR = 2–3) with the same variants (2 L315R, 10 P301L, 6 G272V), and developed a clinicopathological staging model for P301L and G272V variants. The 68-year-old presymptomatic L315R carrier (FTLD-CDR = 0) had limited tau burden morphologically similar to L315R end-stage carriers in middle frontal, antero-inferior temporal, amygdala, (para-)hippocampus and striatum, along with age-related Alzheimer’s disease neuropathological change. The 59-year-old prodromal P301L carrier (FTLD-CDR = 0.5) had highest tau burden in anterior cingulate, anterior temporal, middle/superior frontal, and fronto-insular cortex, and amygdala. The 45-year-old early-stage G272V carrier (FTLD-CDR = 1) had highest tau burden in superior frontal and anterior cingulate cortex, subiculum and CA1. The severity and distribution of tau burden showed some regional variability between variants at presymptomatic/early-stage, while neuronal degeneration, mild-to-moderate, was similarly distributed in frontotemporal regions. Early-stage tau burden and neuronal degeneration were both less severe than in intermediate-/end-stage cases. In a subset of regions (10 GM, 8 WM) used for clinicopathological staging, clinical severity correlated strongly with neuronal degeneration (rho = 0.72, p < 0.001), less strongly with GM tau burden (rho = 0.57, p = 0.006), and did not with WM tau burden (p = 0.9). Clinicopathological staging showed variant-specific patterns of early tau pathology and progression across stages. These unique data demonstrate that tau pathology and neuronal degeneration are present already at the presymptomatic/early-stage of FTLD-MAPT, though less severely compared to intermediate/end-stage disease. Moreover, early pathological patterns, especially of tau burden, differ partly between specific *MAPT* variants.

**Supplementary Information:**

The online version contains supplementary material available at 10.1186/s40478-023-01588-9.

## Introduction

Frontotemporal lobar degeneration (FTLD) comprises a heterogeneous group of neuropathologies characterized by inclusions of either the tau protein (FTLD-Tau), the TDP-43 protein (FTLD-TDP), or less commonly the FET protein family (FTLD-FET) [30]. Familial cases of FTLD-TDP pathologies occur due to defects in the genes including *C9orf72*, *GRN*, *TARDBP* and *VCP*, whereas FTLD-Tau is associated with pathogenic variants in the *MAPT* gene [27].

These neuropathologies are relatively rare, limiting the feasibility of neuropathological studies attempting to identify sites of pathological onset and spread of disease [61]. Staging attempts of FTLD autopsy cohorts stratified based on clinical severity, similar to those performed in Alzheimer’s disease (AD) and Parkinson’s disease [5, 7, 8, 70], have been hindered by the limited availability of tissue and by the pathological heterogeneity of FTLD proteinopathies. Few recent staging attempts based on pure neuropathological data have been performed for FTLD-TDP with behavioural variant frontotemporal dementia (bvFTD) or with amyotrophic lateral sclerosis (ALS) [11, 12], and for subvariants of FTLD-Tau [31, 38], yielding information on hypothesized sequential patterns of pathological involvement. However, these studies exclusively examined patients with relatively advanced clinical disease, and could not verify these hypothetical sequential patterns of pathology in early-stage cases.

Early-stage tissue from FTLD cases is extremely rare. Few studies described early-disease FTLD-TDP pathology in carriers of *C9orf72* repeat expansions [53, 73]. On the other hand, no early-stage pathological cases have been described of genetic FTLD-Tau with *MAPT* pathogenic variants (FTLD-MAPT), to our knowledge. Recently, we have shown that end-stage pathological severity and tau distribution in FTLD-MAPT vary depending on the tau isoform(s) detected in pathological inclusions, which can include predominantly three microtubule-binding repeats (3R) tau, four repeats (4R) tau, or a combination (3R + 4R) [26]. Specific *MAPT* variants also show important variability in pathological features such as cellular involvement and morphological presentations [18, 22, 26]. To further the understanding of FTLD-MAPT disease, the study of early-stage neuropathological features would be highly insightful, shedding light on early patterns of disease onset and spread that may differ across *MAPT* variants. Moreover, the ascertainment of neuropathological changes in the early stage would support the development of tau-specific markers for early clinical detection, such as tau PET tracers [40] or time quaking-induced conversion (RT-QuIC) of tau strains [17], which are currently being developed or optimized.

Having unique access to tissue from three presymptomatic or early-stage *MAPT* variant carriers, we aimed to (1) systematically study the pathological distribution and severity of tau burden and neuronal degeneration in a large set of grey and white matter regions in these carriers, (2) compare them to a larger group of intermediate-/end-stage *MAPT* carriers, and (3) relate these neuropathological features to clinical severity.

## Materials and methods

### Patients

We studied three carriers of *MAPT* pathogenic variants who came to autopsy at presymptomatic stage (1 with L315R variant) or early-stage (1 G272V, 1 P301L). Presymptomatic stage was defined as having no symptoms consistent with FTD prior to death. Early-stage was defined as having mild FTD symptoms, consistent with a global Clinical Dementia Rating plus NACC FTLD [44] (FTLD-CDR) score ≤ 1 immediately prior to autopsy. All three carriers died prematurely of reasons unrelated to FTD. Additionally, we included an intermediate- or end-stage reference cohort with the same pathogenic variants (2 L315R, 10 P301L, 6 G272V). The total cohort consisted of 21 cases who came to autopsy between 2000 and 2020. All symptomatic carriers were under clinical follow-up by a neurologist at the Erasmus University Medical Center, Rotterdam, the Netherlands. The presymptomatic carrier was a first-degree family member of one of the symptomatic carriers, and was not clinically evaluated. Clinical information confirming the absence of cognitive symptomatology was obtained through the Netherlands Brain Bank. All study procedures were performed following local medical ethical regulations, conforming to the Declaration of Helsinki, and informed consent for brain donation was obtained prior to autopsy.

### Clinical data

Available clinical data were collected from clinical charts from Erasmus University Medical Center in a standardized manner by a clinician with expertise on FTD (LAAG) as described [24]. Briefly, we obtained information about clinical features of language, behaviour, motor and other cognitive domains. For symptomatic carriers, the clinical diagnosis was determined based on baseline clinical data (within 3 years of disease onset), gathered from the clinical history and bedside neurologic examinations, according to current clinical criteria [54]. Additionally, FTLD-CDR scores prior to death were retrospectively scored by two independent raters (HS, LAAG) based on detailed clinical data from clinical charts. These scores enabled stratification of the cohort into presymptomatic/early-stage (FTLD-CDR ≤ 1), intermediate-stage (FTLD-CDR = 2) and end-stage (FTLD-CDR = 3).

### Neuropathological diagnosis

Brain autopsy was carried out according to the Legal and Ethical Code of Conduct of the Netherlands Brain Bank. At the Netherlands Brain Bank, tissue collected at autopsy from one hemisphere was fixed in 10% neutral buffered formalin for four weeks and subsequently embedded in paraffin blocks. Per protocol, paraffin-embedded tissue was cut into 6 μm sections for haematoxylin-eosin (H&E) stain, Bodian’s silver stain, and immunohistochemistry for tau, Aβ, TDP-43 and alpha-synuclein with well-characterized antibodies. Neuropathological diagnosis was established by an expert neuropathologist at the Netherlands Brain Bank (AJMR) using established criteria [37, 43, 46].

### Genetic analysis

All cases were screened and tested positively for a pathogenic genetic variant in the *MAPT* gene, with the exception of one end-stage case who did not undergo genetic testing, but came from a family carrying the L315R variant and had clinical and pathological features typical of this variant.

### Brain sampling

Brain regions were selected based on knowledge of affected regions from studies of antemortem atrophy patterns in presymptomatic [15, 51, 68] and symptomatic [55, 77] *MAPT* variant carriers. We examined nine cortical grey matter (GM) regions and their adjacent juxtacortical white matter (WM): anterior cingulate cortex (ACC, Brodmann area [BA] 24), anterior temporal cortex (ATC, BA 38), entorhinal cortex (EC, BA 28), fronto-insular cortex (FIC), fusiform gyrus (FG, BA 36), inferior parietal lobule (IPL, BA 39), middle frontal cortex (MFC, BA 46), transentorhinal cortex (TEC, BA 35), medial superior frontal cortex (SFC, BA 9). The ACC and SFC were two adjacent gyri sampled from the same tissue slide (cingulate tissue block). The EC, TEC and FG were all sampled from the same tissue slide (mid-hippocampal tissue block). The FIC was sampled from the striatal tissue block as the cortex lateral to the claustrum, and was only available in a subset of cases (FIC = 16). Additionally, we included three subcortical GM regions that have been implicated in FTLD-MAPT: amygdala, hippocampus and striatum. We sampled the amygdala as a single structure (AMY), while in the hippocampus we distinguished hippocampal subfields of dentate gyrus (DG), cornu ammonis (CA) 1–4 and subiculum (SUB), and in the striatum we identified the caudate nucleus (CAU) and putamen (PUT). In total, we examined nine subcortical GM (sub-)regions. Finally, four subcortical WM regions approximating WM tracts were sampled if deep WM (below the sulcal depths) was available in a tissue section, i.e. the anterior superior longitudinal fasciculus (aSLF) in the MFC section, the posterior superior longitudinal fasciculus (pSLF) in the IPL section, the corpus callosum (CC) in the ACC section and the internal capsule (IC) in the striatal section. Tissue was sampled from the right hemisphere, as per protocol at the Netherlands Brain Bank, in all cases except for one (1 early-stage G272V case), who was sampled primarily in the left hemisphere, but had bilateral sampling for the hippocampus and middle frontal tissue blocks.

### Immunohistochemistry and digital histopathology

Immunohistochemistry was performed using anti-phospho tau antibody (AT8, #MN1020, Invitrogen; dilution 1:400; DAB incubation time 3 min) as primary antibody to detect phosphorylated tau on 6-µm thick slides.

Using QuPath software (version 0.3.2), through validated digital histopathology methods previously described [23, 24, 32, 33], AT8-positive tau pathology was quantified as the percentage of area occupied (%AO) by tau positive-pixels in GM and WM regions of interest (ROI). These digital methods are less time-intensive than unbiased stereology, enabling the acquisition of quantified pathological data from a large number of regions [33]. Briefly, cortical GM ROIs were obtained using a transect-belt sampling method as the longest stretch of relatively flat cortex within a specific neuroanatomical region to avoid bias from overrepresentation of a subset of cortical layers [2]. Additionally, WM ROIs were obtained as a rectangular or polygonal annotation adjacent to the cortical GM ROI (i.e. juxtacortical WM) and outside the sulcal depths (i.e. subcortical WM), where available. Next, to reduce sampling bias, the mean from a random sample of 175 × 175 μm tiles occupying 30% of each ROI was used to generate the %AO measurement as described [32, 33]. In subcortical GM/WM areas, the entire anatomical region of interest was annotated. Hippocampal subfields were distinguished based on cytoarchitectural features of the layers and anatomical landmarks (Suppl. Figure 1), as previously done [1], with the help of the Allen Human Reference atlas (https://atlas.brain-map.org/) to verify the sectioning level of the tissue and identify the correct anatomical structures. All subcortical sampling was independently reviewed by two investigators and minor discrepancies in sampling were resolved through consensus (LAAG, RB, SA).

The %AO by tau pathology in each ROI was measured using empirically derived RGB detection algorithms for each staining batch and optical density (OD) thresholds, which enable to account for potential batch variation [23]. Digital histopathological parameters were tested and optimized based on visual inspection of a set of 4–5 slides from the same staining batch as described [33]. Intra-individual variability was accounted for by staining all slides from the same case within the same batch. Inter-individual variability was accounted for statistically using mixed modelling (see Statistical Analysis below).

Digital scores of %AO by tau pathology were validated by comparison to traditional ordinal scores (i.e. 0–3), obtained blinded to quantitative pathology measurements as previously described [31] (Suppl. Figure 2). Our total dataset consisted of 173 cortical GM, 162 subcortical GM, 171 juxtacortical WM and 68 subcortical WM %AO measurements (total 574), obtained from 120 tissue slides. Missing data and damaged tissue were excluded from the analyses. We provide an overview of all available %AO measurements in Suppl. Table 1.

### Neuronal degeneration assessment

Cortical and subcortical GM regions were assessed systematically using an ordinal scale for neuronal degeneration comprising several interrelated parameters, i.e. neuronal density, vacuolation and cortical lamination (for isocortical and proisocortical regions only), as described [26]. Briefly, score 0 corresponded to normal cortex, while scores 1–4 corresponded to mild, moderate, severe and very severe neuronal degeneration, respectively. Neuronal degeneration scores (NDS) were assigned blinded to *MAPT* genetic variant and tau pathology, based on haematoxylin counterstain of AT8-stained slides (with DAB channel off in QuPath). All sections were scored by two independent raters (AR, LAAG, RB) using this scale, and in case discrepancies were present, scores were discussed to achieve consensus.

### Statistical analysis

Due to the small sample size of the cohort (especially the early-stage carriers), some of the results presented are purely descriptive. Statistics was applied to analyses across the entire cohort (i.e. correlations between clinical stages and pathological severity) or comparing clinical stage groupings of carriers (i.e. early- vs. intermediate- vs. end-stage) across multiple regions. Analyses of tau burden (i.e. %AO by tau pathology) and neuronal degeneration were performed using mixed modelling, either linear or ordinal as appropriate, to account for multiple measurements from the same individual and for missing data. %AO scores were transformed through natural logarithm transformation to meet the assumptions of the models. Correlation analyses of FTLD-CDR score with pathological severity (i.e. tau burden, neuronal degeneration) were performed using Spearman’s correlation, per region or taking the median of multiple regions. All analyses were performed using R statistical software 4.2.2, with significance level set at 0.05.

## Results

### Clinical and demographic characteristics

The presymptomatic L315R carrier (FTLD-CDR = 0) did not have any cognitive, behavioural or motor symptoms and died at age 68 due to the consequences of lung cancer. The prodromal P301L carrier (FTLD-CDR = 0.5) had subjective cognitive complaints at age 48 (11 years prior to autopsy), but these could not be objectified through formal neuropsychological examination. At age 50, a SPECT scan showed mild decreased perfusion of the frontal cortex. In the following years, she experienced depressive symptoms, tremor and loss of strength in the right extremities but no cognitive decline. She was diagnosed with metastasized lung cancer at age 58, and one year later she underwent euthanasia. Before death, she had (very) mild symptoms, i.e. word-finding difficulties and loss of initiative, consistent with a diagnosis of mild cognitive and/or behavioural and/or motor impairment (MCBMI) [4]. However, she was not seen clinically at our cognitive clinic, so the diagnosis could not be formally ascertained. The early-stage G272V carrier (FTLD-CDR = 1) noticed the first behavioural changes at age 39, which were considered primarily associated with depressive symptoms and a manic-psychotic episode. Cognitive decline was ascertained through neuropsychological testing at age 43. Around the same time, an FDG-PET scan showed focal right frontal hypometabolism, while no significant change was detected on structural MRI. Mild right-lateralized frontotemporal atrophy was observed only one year later. He was diagnosed with bvFTD and, because of this diagnosis and related future perspective, he chose to undergo euthanasia at age 45.

The intermediate-stage group included 2 P301L and 1 G272V carriers. All cases had been diagnosed with bvFTD and died relatively early due to sudden cardiac arrest (n = 2) and gastrointestinal bleeding (n = 1), while the dementia severity was still moderate (FTLD-CDR = 2). Median disease duration was 3 years (range 2–7 years).

The end-stage group included 8 P301L, 5 G272V and 2 L315R carriers. All cases had a clinical diagnosis of bvFTD and were severely affected before death (FTLD-CDR = 3). Median disease duration was 8 years (range 4–20 years). Further demographic and autopsy features can be found in Table 1.

**Table 1:** Demographic and clinical features of the cohort

Case	*MAPT* variant	*MAPT* isoform	Sex	Age onset (yrs)	Age death (yrs)	Disease duration (yrs)	Group	FTLD-CDR	Clinical diagnosis	PMI (hrs)	Brain weight (g)	Co-pathology
1	L315R	3R + 4R	F		68		Early-stage	0		6	1188	Age-related tau (B2)
2	P301L	4R	F		59		Early-stage	0.5	MCBMI^a^	6	1079	
3	G272V	3R	M	43	45	2	Early-stage	1	bvFTD	6	1495	
4	P301L	4R	M	49	52	3	Intermediate-stage	2	bvFTD	12	1115	
5	P301L	4R	M	53	55	2	Intermediate-stage	2	bvFTD	4	1100	
6	G272V	3R	F	47	54	7	Intermediate-stage	2	bvFTD	8	1027	
7	L315R	3R + 4R	F	54	63	9	End-stage	3	bvFTD	4	758	
8	L315R	3R + 4R	M	52	57	5	End-stage	3	bvFTD	8	1111	
9	P301L	4R	M	58	66	8	End-stage	3	bvFTD	5	1011	
10	P301L	4R	M	39	46	7	End-stage	3	bvFTD	6	1065	
11	P301L	4R	F	56	66	10	End-stage	3	bvFTD	7	707	
12	P301L	4R	M	51	60	9	End-stage	3	bvFTD	5	887	
13	P301L	4R	F	50	64	14	End-stage	3	bvFTD	8	652	
14	P301L	4R	M	52	64	12	End-stage	3	bvFTD	6	1029	
15	P301L	4R	M	53	57	4	End-stage	3	bvFTD	5	1197	Vascular (infarcts)^b^
16	P301L	4R	M	57	65	8	End-stage	3	bvFTD	6	1002	
17	G272V	3R	F	45	54	9	End-stage	3	bvFTD	6	791	
18	G272V	3R	F	47	67	20	End-stage	3	bvFTD	4	757	
19	G272V	3R	M	41	49	8	End-stage	3	bvFTD	5	1188	Beta-amyloid (A1, C0)
20	G272V	3R	M	42	51	9	End-stage	3	bvFTD	4	962	Vascular (ischemic scars)^c^
21	G272V	3R	M	42	49	7	End-stage	3	bvFTD	5	1105	

Legend: bvFTD = behavioural variant frontotemporal dementia; FTLD-CDR = global Clinical Dementia Rating plus FTLD NACC; MCBMI = mild cognitive and/or behavioural and/or motor impairment; PMI = postmortem interval

^a^Only mild symptoms were present immediately before death, likely consistent with an MCBMI diagnosis. As the patient was not seen clinically during that period, the diagnosis could not be formally ascertained, and the exact age of symptom onset is uncertain (between 50–55 years of age)

^b^Case 15 showed two small old cortical infarctions in the motor cortex and one small recent cortical infarction in the occipital cortex

^c^Case 20 had small ischemic scars in the occipital cortex

### Pathology of the presymptomatic L315R carrier

**Fig. 1:**
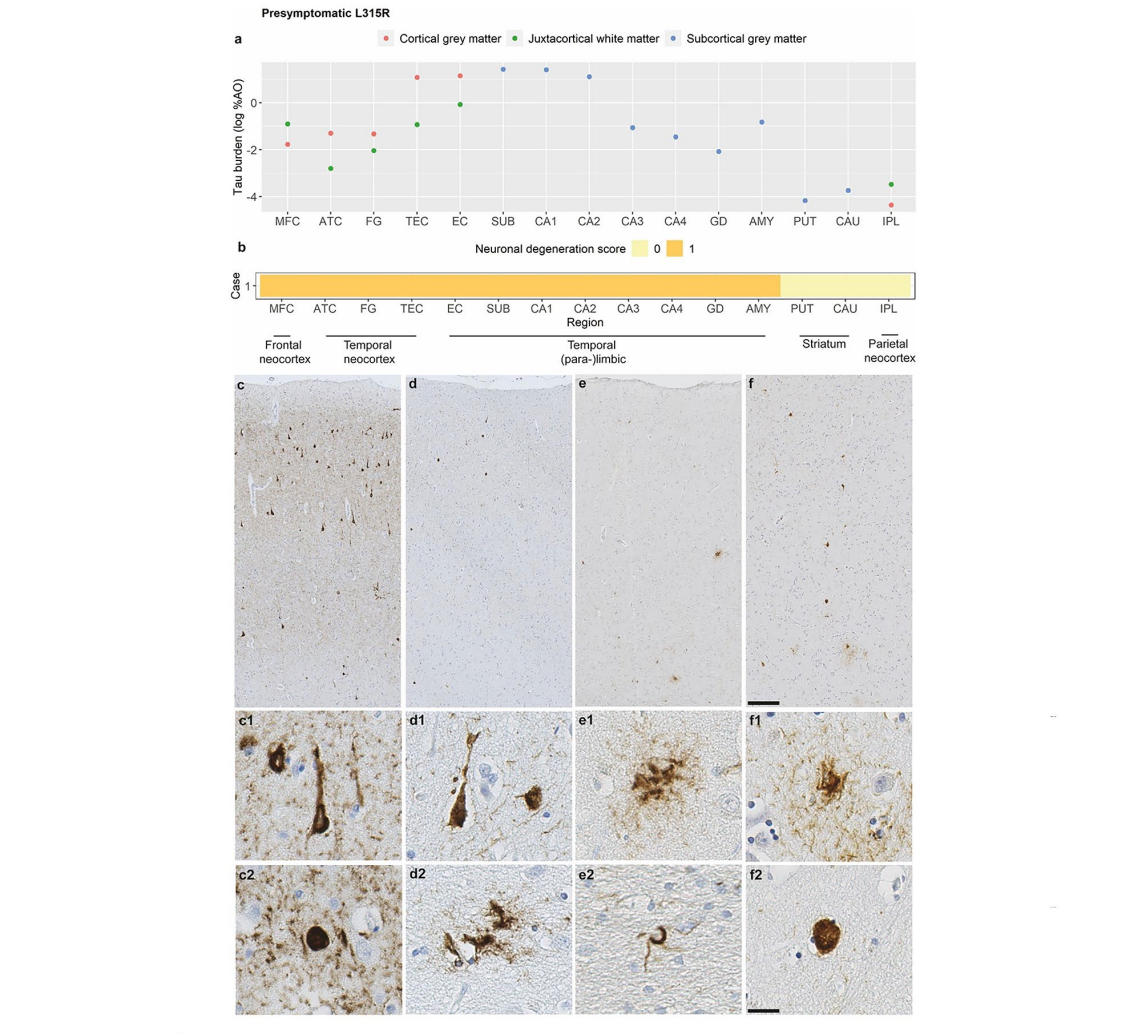
Pathological features of the presymptomatic L315R carrier Figure portrays the severity of tau burden in grey matter and white matter regions **(a)**, the severity of neuronal degeneration per region **(b)**, and the morphology of neuropathological tau inclusions **(c-f)** in the presymptomatic L315R case. The transentorhinal cortex **(c)** and other temporal regions showed age-related tau pathology (A0B2C0), appearing as Alzheimer-like argyrophilic neurofibrillary tangles **(c1)** and neuritic threads, but also Pick-body like inclusions **(c2)** characteristic of the L315R variant. The anterior temporal cortex **(d)** was characterized by milder age-related tau pathology **(d1)** and seldom a Pick body-like inclusion, but also astrocytic inclusions consistent with L315R pathology **(d2)**. Similar ramified **(e1)** or punctate **(f1)** astrocytes were found in the middle frontal cortex **(e)** and amygdala **(f)**, together with oligodendrocytic coiled bodies in the middle frontal white matter **(e2)** and Pick body-like inclusions **(f2)** in the amygdala. Scale overview = 200 μm; scale magnification = 20 μm Legend: %AO = percentage of area occupied (by tau-positive pixels); AMY = amygdala; ATC = anterior temporal cortex; CA1-4 = cornu ammonis 1–4; CAU = caudate nucleus; EC = entorhinal cortex; FG = fusiform gyrus; GD = gyrus dentatus; IPL = inferior parietal lobule; MFC = middle frontal cortex; PUT = putamen; SUB = subiculum; TEC = transentorhinal cortex Tau burden (y-axis, a) corresponds to the digitally measured %AO by tau pathology after applying natural logarithmic transformation

The presymptomatic L315R carrier showed highest tau burden in medial temporal regions (Fig. 1a), which was in part consistent with age-related tau pathology without co-occurring beta-amyloid pathology (A0B2C0). Alzheimer-like argyrophilic neurofibrillary tangles and neuropil threads were present throughout the transentorhinal (TEC; Fig. 1c) and entorhinal cortex (EC), hippocampal subfields (especially subiculum [SUB], CA1-2), and to a lesser extent in the antero-inferior temporal cortex (fusiform gyrus [FG], anterior temporal cortex [ATC]; Fig. 1d). In addition to age-related tau pathology, few neuronal Pick body-like inclusions, characteristic of the L315R variant [26, 72], were observed in these temporal neocortical and (para-)limbic regions (Fig. 1c-d), as well as in the middle frontal cortex (MFC; Fig. 1e), amygdala (AMY; Fig. 1f), and striatum. Additionally, ramified and punctate astrocytic tau inclusions consistent with L315R pathology [26] were present in the MFC, AMY, ATC, SUB and CA1, often located in GM, especially at the GM-WM junction, and in juxtacortical WM (Fig. 1d-f). The middle frontal and parahippocampal gyri also showed coiled body-like inclusions in the WM (Fig. 1e). There was mild neuronal degeneration throughout the frontotemporal neocortex (MFC, ATC, FG, TEC), and (para-)limbic regions (EC, SUB, CA1-4, GD and AMY). Other regions were still relatively spared (NDS = 0; Fig. 1b). In terms of laminar distribution, neuronal tau pathology and neuronal degeneration were more pronounced in laminae II-III, especially in temporal regions, whereas astrocytic tau pathology was predominantly observed at the GM-WM junction, in lamina VI and in part V (while sporadically in upper laminae).

### Pathology of the prodromal P301L carrier

**Fig. 2:**
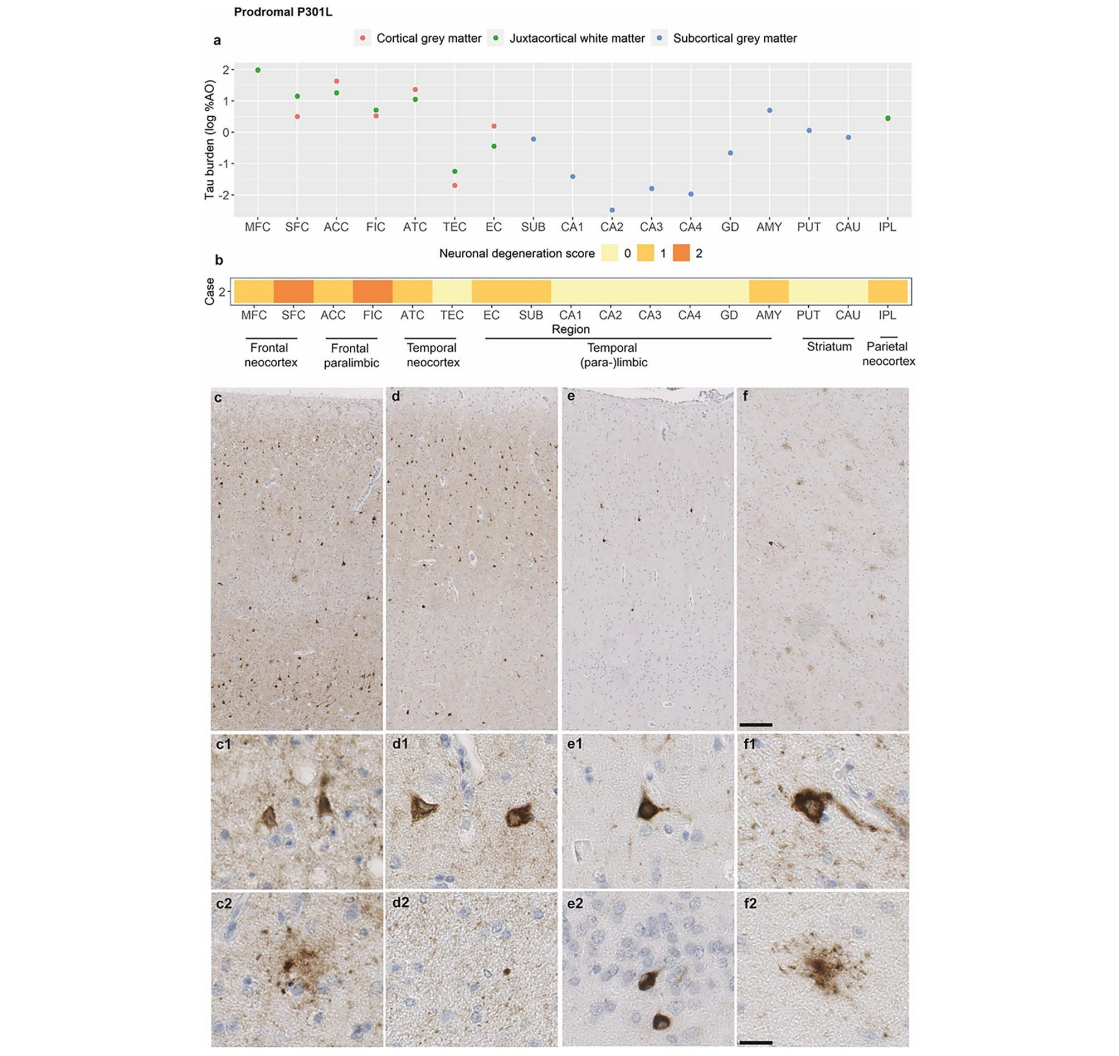
Pathological features of the prodromal P301L carrier Figures portrays the severity of tau burden in grey matter and white matter regions **(a)**, the severity of neuronal degeneration per region **(b)**, and the morphology of neuropathological tau inclusions **(c)** in the prodromal P301L case. There was moderate neuronal tau pathology in frontal neocortical/paralimbic and temporal neocortical regions, such as the middle frontal cortex **(c)** and the anterior temporal cortex **(d)**. Inferior parietal, (para-)hippocampal regions such as the transentorhinal cortex **(e)**, and the striatum **(f)** were more mildly affected. Neuronal pathology appeared primarily as diffuse neuronal cytoplasmic inclusions and neurofibrillary-tangle like inclusions **(c1, e1)**, perinuclear rings **(d1, f1)**, or round compact inclusions the dentate gyrus **(e2)**. Mild amounts of globular astrocytic inclusions **(c2)**, and less often granular-fuzzy astrocytes **(f2)**, were present. White matter pathology was limited, and appeared as white matter threads and sporadically as a globular oligodendrocytic inclusion **(d2)**. Scale overview = 200 μm; scale magnification = 20 μm Legend: %AO = percentage of area occupied (by tau-positive pixels); ACC = anterior cingulate cortex; AMY = amygdala; ATC = anterior temporal cortex; CA1-4 = cornu ammonis 1–4; CAU = caudate nucleus; EC = entorhinal cortex; FG = fusiform gyrus; FIC = fronto-insular cortex; GD = gyrus dentatus; IPL = inferior parietal lobule; MFC = middle frontal cortex; PUT = putamen; SFC = superior frontal cortex; SUB = subiculum; TEC = transentorhinal cortex Tau burden (y-axis, a) corresponds to the digitally measured %AO by tau pathology after applying natural logarithmic transformation

The prodromal P301L carrier showed highest tau burden in the MFC, ACC and ATC (Fig. 2a). Neuronal pathology appeared predominantly as perinuclear rings, diffuse neuronal cytoplasmic inclusions and neurofibrillary-tangle like inclusions, and was moderate in frontal neocortical/paralimbic (MFC, SFC, ACC; Fig. 2c) and temporal neocortical/limbic regions (ATC, AMY; Fig. 2d). There was milder pathology in the IPL, striatum and hippocampal regions (TEC, EC, SUB, CA1, GD; Fig. 2e), while CA2-4 were relatively spared. Astrocytic pathology, predominantly as globular astrocytic inclusions and less often as granular-fuzzy astrocytes, was especially present in the striatum (moderate severity; Fig. 2f), IPL and MFC (both mild severity). Oligodendrocytic intracellular pathology, as small globular inclusions, was minimal, while most regions showed mild-to-moderate diffuse WM threads. There was mild-to-moderate neuronal degeneration in frontal neocortical (MFC, SFC) and paralimbic (ACC, FIC) regions, as well as mild degeneration in the temporal neocortex (ATC), (para-)limbic cortex (EC, SUB, AMY) and inferior parietal cortex (IPL; Fig. 2b). In cortical regions, tau inclusions were observed in both upper (II-III) and lower (V, VI to a lesser extent) laminae, while neuronal degeneration was more pronounced in laminae II-III.

### Pathology of the early-stage G272V carrier

**Fig. 3:**
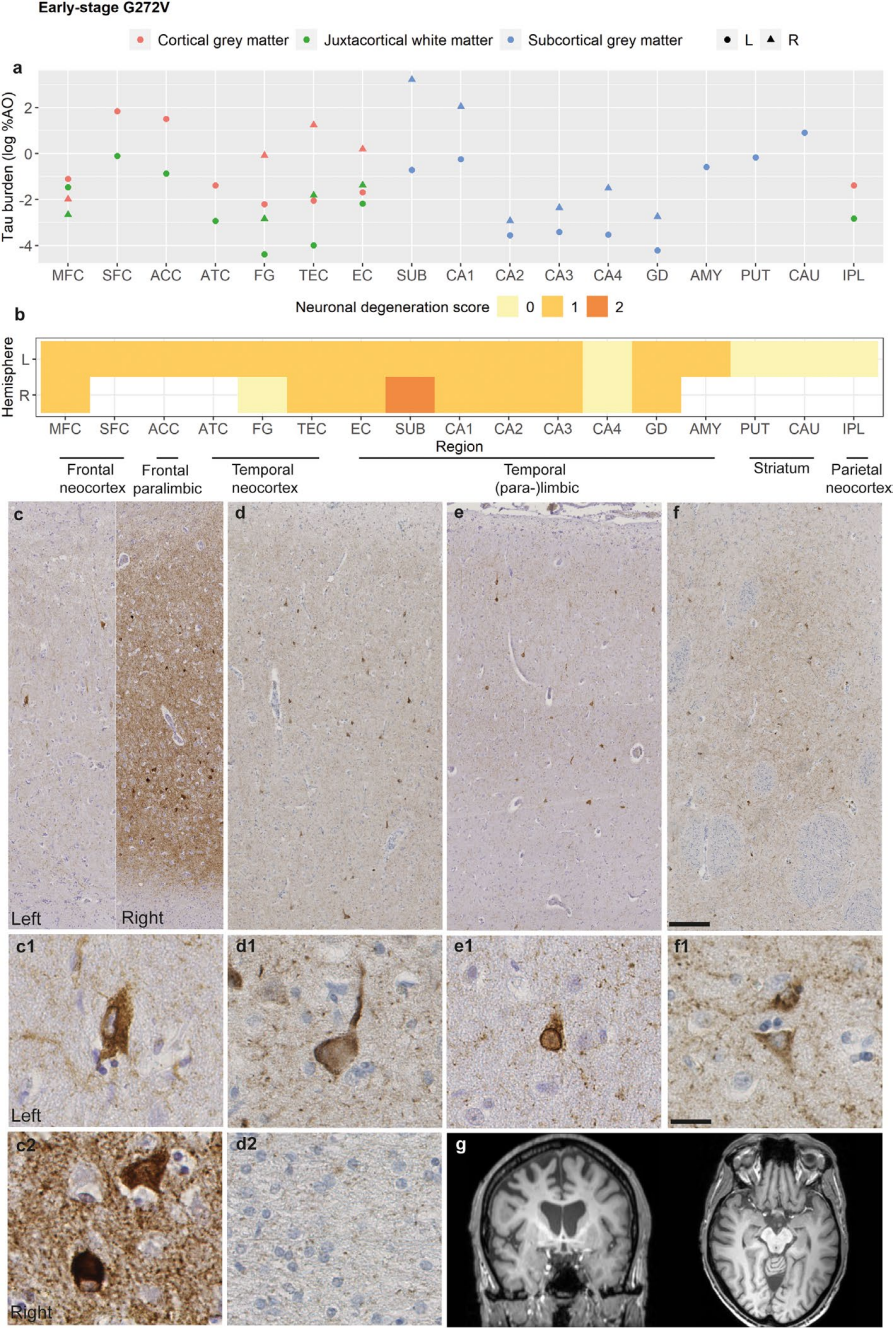
Pathological features of the early-stage G272V carrier Figure portrays the severity of tau burden in grey matter and white matter regions **(a)**, the severity of neuronal degeneration per region **(b)**, and the morphology of neuropathological tau inclusions **(c-f)** in the early-stage G272V case. Moderate tau pathology was observed in frontotemporal neocortical and paralimbic regions, such as the subiculum **(c)**, the superior frontal cortex **(d)**, and the anterior temporal cortex **(e)**. The striatum **(f)** was also moderately affected. Left-hemisphere regions were generally less affected than right-hemisphere regions (**c**; however, only hippocampus and middle frontal cortex were available from the right hemisphere), which was also consistent with evidence of right-lateralized atrophy from antemortem imaging **(g)**. Tau pathology was predominantly neuronal, appearing as diffuse neuronal cytoplasmic inclusions **(c1, f1)**, neurofibrillary tangle-like inclusions **(c2)**, neuronal grains, and less often as Pick body-like inclusions **(c2)**, ballooned neurons **(d1)** and perinuclear rings **(e1)**. The white matter showed tiny diffuse threads and no intracellular oligodendrocytic pathology **(c2)**. Scale overview = 200 μm; scale magnification = 20 μm Legend: %AO = percentage of area occupied (by tau-positive pixels); ACC = anterior cingulate cortex; AMY = amygdala; ATC = anterior temporal cortex; CA1-4 = cornu ammonis 1–4; CAU = caudate nucleus; EC = entorhinal cortex; FG = fusiform gyrus; GD = gyrus dentatus; IPL = inferior parietal lobule; MFC = middle frontal cortex; PUT = putamen; SFC = superior frontal cortex; SUB = subiculum; TEC = transentorhinal cortex Tau burden (y-axis, a) corresponds to the digitally measured %AO by tau pathology after applying natural logarithmic transformation

The early-stage G272V carrier had evidence of largely right-lateralized frontotemporal atrophy on antemortem MRI (interval MRI-autopsy = 0.1 years; Fig. 3g), with frontal neocortical (SFC, MFC), paralimbic (ACC), temporal neocortical (ATC), hippocampal and striatal (PUT) involvement (Suppl. Figure 3). In postmortem pathology, right-hemisphere hippocampal subregions (SUB, CA1; Fig. 3a) showed the highest tau burden, higher than the left-hemisphere ones, and were followed by left-hemisphere frontal neocortical and paralimbic regions (SFC, ACC), anterior temporal regions (ATC), striatum and amygdala. Left-hemisphere medial and inferior temporal regions (SUB, CA1, TEC, EC, FG) were more mildly affected, while IPL, MFC, and other hippocampal subregions (CA2-4, GD) had even milder tau burden. Tau burden in MFC was similar in both hemispheres. Neuronal tau pathology consisted of diffuse neuronal cytoplasmic inclusions (Fig. 3c and f), neurofibrillary tangle-like inclusions (Fig. 3c), and less often of Pick body-like inclusions (Fig. 3c), perinuclear rings (Fig. 3e) and ballooned neurons (Fig. 3d). Additionally, neuronal grains were mildly-to-moderately abundant. Astrocytic inclusions were sporadic. The GM was consistently more affected than the WM, which only showed tiny diffuse WM threads and no intracellular oligodendrocytic pathology (Fig. 3d). Mild-to-moderate neuronal degeneration was observed in the frontotemporal neocortex (MFC, SFC, ATC, FG, TEC) and (para-)limbic regions (ACC, EC, SUB, CA1-3, GD, AMY; Fig. 3b), and did not show lateralization in regions with bilateral tissue (hippocampus and MFC) except for a slightly greater involvement of the right SUB. In cortical regions, tau pathology and neuronal degeneration were slightly more pronounced in laminae II-III, but tau pathology extended to the lower laminae (V, VI to a lesser extent), too, even in less affected cortical regions (IPL). This carrier also showed histological signs of immune activation and infiltration, described in detail elsewhere [74], consistent with a concomitant probable diagnosis of (autoimmune) encephalitis. Routine antemortem CSF diagnostics for immune-mediated encephalitis was negative.

### Comparison of early pathology between *MAPT* variants

**Fig. 4:**
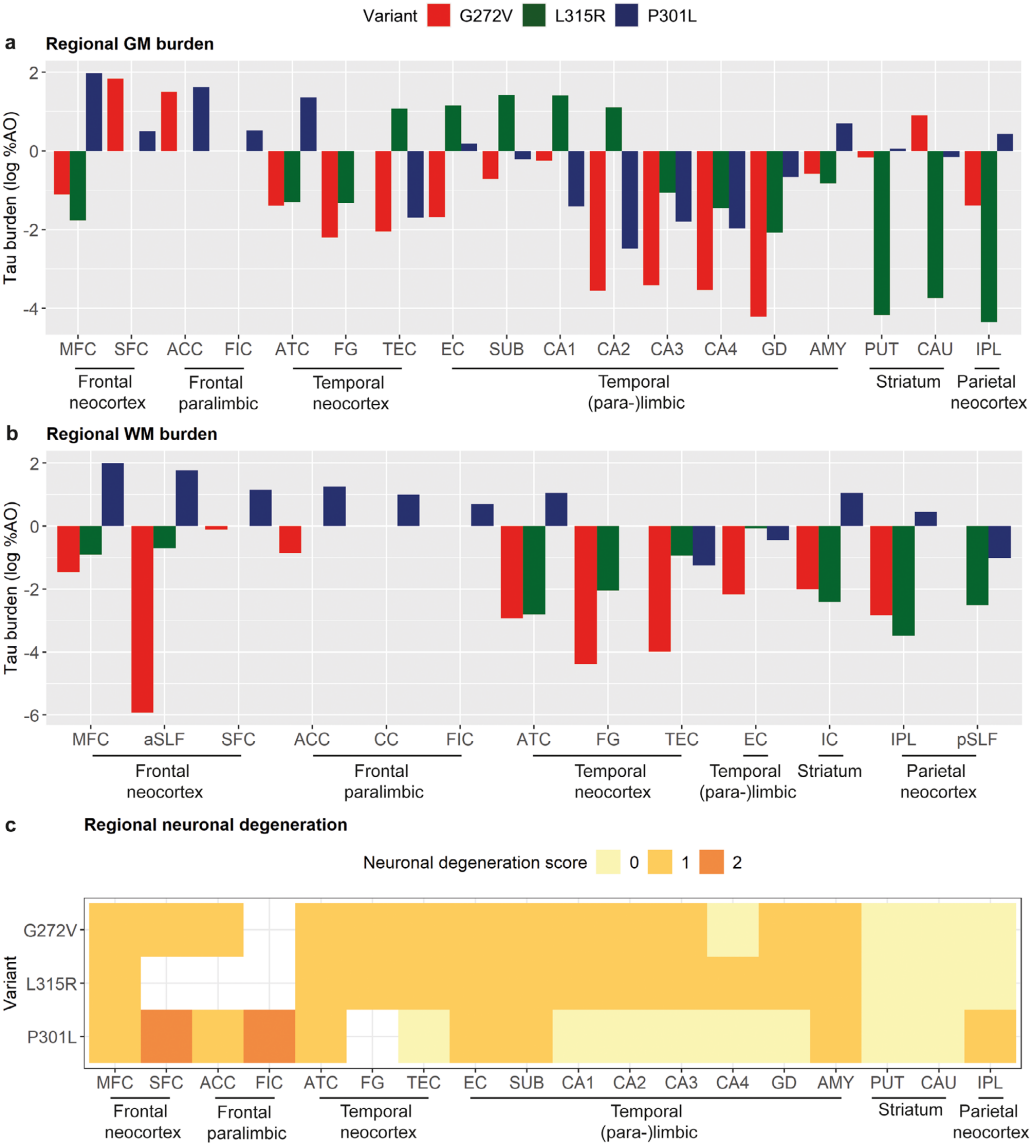
Comparisons of presymptomatic/early-stage pathological features between the three *MAPT* variants Plots portray the severity of grey matter tau burden **(a)**, white matter tau burden **(b)** and neuronal degeneration **(c)** in the three early cases (1 presymptomatic L315R, 1 prodromal P301L and 1 early-stage G272V). The regional distribution of early tau burden differed between different variants **(a-b)**. In contrast, neuronal degeneration was of similar severity and quite similar regional distribution, with few exceptions (medial temporal sparing and mild inferior parietal involvement in the P301L carrier; **c**). Data from only one brain hemisphere are shown for each case (L315R right, P301L right, G272V left) Legend: %AO = percentage of area occupied (by tau-positive pixels); AMY = amygdala; ACC = anterior cingulate cortex; aSLF = anterior superior longitudinal fasciculus; ATC = anterior temporal cortex; CA1-4 = cornu ammonis 1–4; CAU = caudate nucleus; CC = corpus callosum; EC = entorhinal cortex; FIC = fronto-insular cortex; FG = fusiform gyrus; GD = gyrus dentatus; IC = internal capsule; IPL = inferior parietal lobule; MFC = middle frontal cortex; pSLF = posterior superior longitudinal fasciculus; PUT = putamen; SFC = superior frontal cortex; SUB = subiculum; TEC = transentorhinal cortex Tau burden (y-axis, a-b) corresponds to the digitally measured %AO by tau pathology after applying natural logarithmic transformation

The three early-stage *MAPT* variant carriers showed a variable distribution of tau burden in GM (Fig. 4a) and WM regions (Fig. 4b). For GM, the L315R carrier showed a pronounced medial temporal distribution of tau pathology (TEC, EC, SUB, CA1-2); the G272V carrier showed prominent fronto-limbic and striatal tau pathology (SFC, ACC, CAU) in the left hemisphere (shown in Fig. 4; limited right-hemisphere data not included in these comparisons); the P301L carrier showed prominent tau pathology with a frontal and anterolateral temporal distribution (MFC, ACC, ATC). For WM, the P301L carrier had overall more severe WM tau pathology compared to the other early carriers, pronounced in both frontotemporal juxtacortical WM (MFC, SFC, ACC, ATC) and subcortical WM tracts (aSLF, CC, IC); the G272V had highest WM burden in a frontal region (SFC) and the L315R in a temporal region (EC), but these were little affected compared to the P301L carrier. Finally, neuronal degeneration was mild or moderate at most across all three early carriers and distributed in a similar manner, involving primarily frontotemporal regions. There was, however, relative sparing of medial temporal regions (TEC, CA1-4, GD) with mild degeneration of the IPL in the P301L carrier (Fig. 4c).

### Comparison of pathological severity between clinical stage groups

**Fig. 5:**
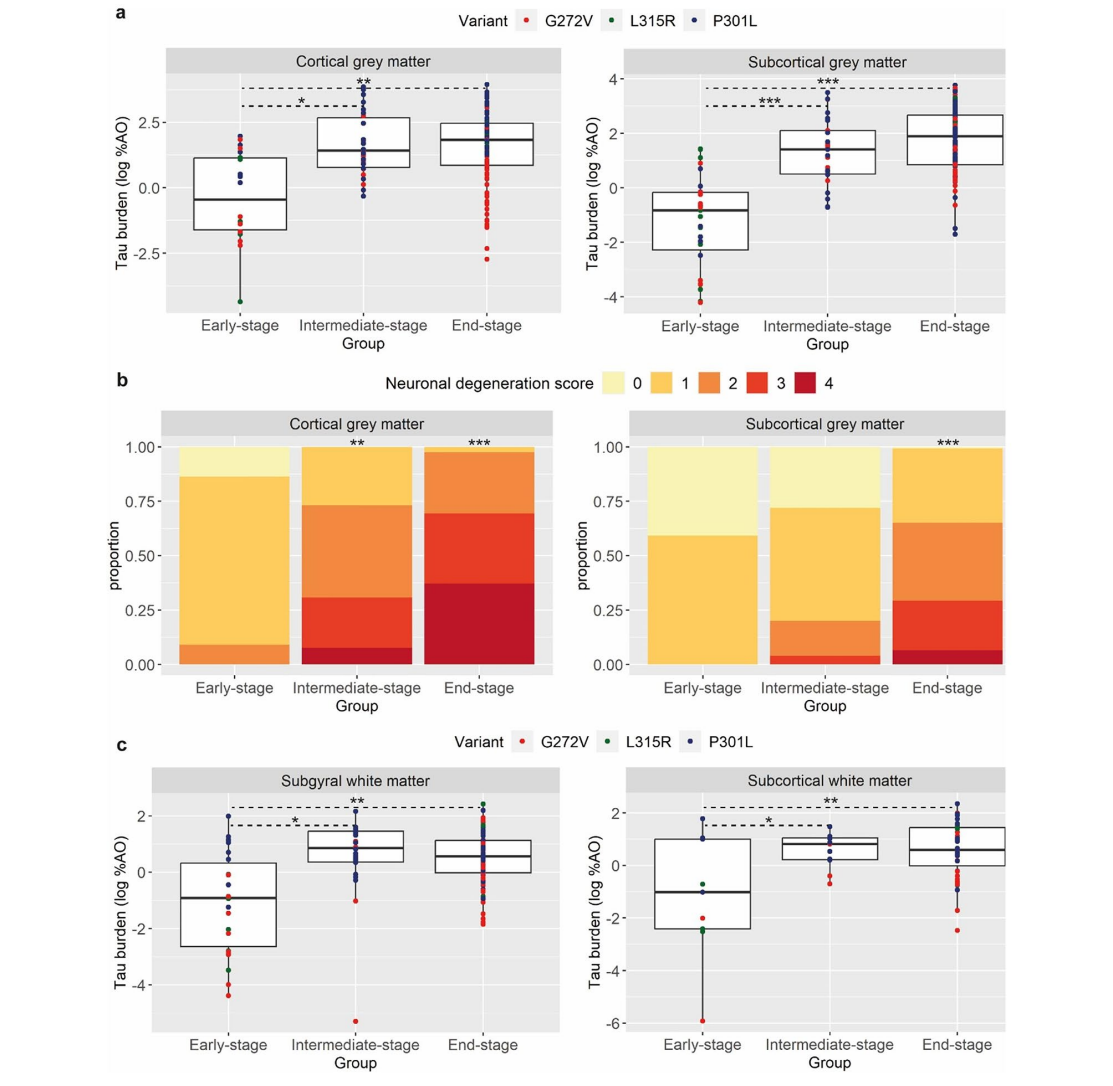
Comparisons of pathological features across clinical stages Plots portray cortical/subcortical grey matter tau burden **(a)**, cortical/subcortical neuronal degeneration **(b)** and juxtacortical/subcortical white matter tau burden **(c)** across clinical stages, independently of region and *MAPT* variant. The early-stage group had lower tau burden compared to intermediate- and end-stage groups in cortical (*p* ≤ 0.021) and subcortical (*p* < 0.001) grey matter, as well as in juxtacortical (*p* ≤ 0.010) and subcortical (*p* ≤ 0.013) white matter. Neuronal degeneration was less severe in the early-stage group compared to intermediate- (*p* = 0.001) and end-stage (*p* < 0.001) in cortical regions, and compared to end-stage only in subcortical grey matter regions (*p* < 0.001) Legend: %AO = percentage of area occupied (by tau-positive pixels) Tau burden (y-axis, a, c) corresponds to the digitally measured %AO by tau pathology after applying natural logarithmic transformation Reported p-values were obtained using linear mixed modelling for tau burden or ordinal mixed modelling for neuronal degeneration score *p < 0.05 compared to the early-stage group **p < 0.01 compared to the early stage group ***p < 0.001 compared to the early-stage group

Next, we compared pathology burden between early-stage (FTLD-CDR ≤ 1), intermediate-stage (FTLD-CDR = 2) and late-stage (FTLD-CDR = 3) groups across all three variants. As expected, we found significantly lower tau burden in cortical and subcortical GM (Fig. 5a) and in juxtacortical and subcortical WM (Fig. 5c) in the early-stage group compared to intermediate- and end-stage groups (p ≤ 0.02 vs. intermediate-stage, p ≤ 0.006 vs. end-stage). Similarly, neuronal degeneration in cortical GM regions was less severe in the early-stage group compared to intermediate- (p = 0.001) and end-stage (p < 0.001) groups, while neuronal degeneration in subcortical GM regions was significantly less severe in the early-stage group compared to the end-stage group only (p < 0.001).

These patterns of tau pathological progression from early- to end-stage varied to some extent in different variants (Suppl. Figures 4–5). In particular, in the G272V variant tau burden was highest at intermediate-stage, while at end-stage a clear decrease in tau burden was observed, especially in GM, more pronounced compared to other variants (Suppl. Figure 4). On the other hand, the P301L variant was characterized by early-affected juxtacortical and subcortical WM, in which tau burden was relatively high already at the early stage (Suppl. Figure 4). As for neuronal degeneration, we observed that the progression to (very) severe neuronal degeneration through early/intermediate clinical stages was more frequent in the G272V variant compared to the P301L variant, especially in cortical GM (Suppl. Figure 5).

### Correlation of regional pathology and clinical severity

For the staging analysis, we focused on five cortical GM regions (ACC, MFC, ATC, TEC, IPL), with their adjacent juxtacortical WM, and five subcortical GM regions (SUB, CA1, GD, AMY, CAU), which were representative of brain areas of interest for FTLD-MAPT and had sufficient amounts of data. For WM staging, we also included three subcortical WM regions (aSLF, pSLF, IC) to assess the relative involvement of juxtacortical vs. subcortical WM. Median GM tau burden across all these regions correlated moderately with FTLD-CDR score (rho = 0.57, p = 0.006), while neuronal degeneration correlated strongly with it (rho = 0.72, p < 0.001). Conversely, median WM tau burden did not correlate with FTLD-CDR score (p = 0.905). Sub-analyses per region found similar results (Suppl. Table 2). Neuronal degeneration in all regions correlated positively with FTLD-CDR score even after correcting for multiple comparisons, except for SUB and AMY. Tau burden in GM MFC, IPL, GD, AMY and CAU, and in WM IPL and IC, correlated weakly with FTLD-CDR score, but these correlations were not significant after correction for multiple comparisons.

### Staging model per variant

**Fig. 6:**
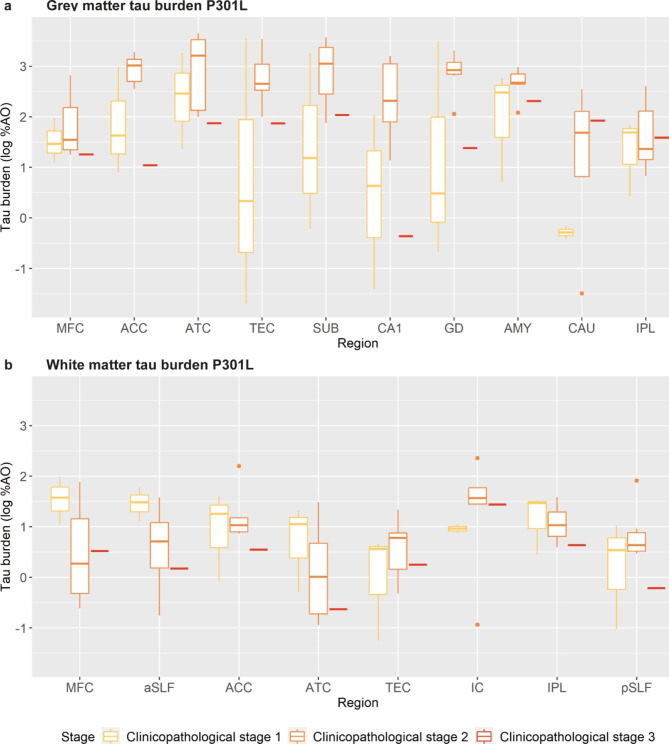
Staging of grey and white matter tau pathology across clinicopathological stages in the P301L variant Plots portray regional progression of grey **(a)** and white **(b)** matter pathology across clinicopathological stages in the P301L variant Legend: %AO = percentage of area occupied (by tau-positive pixels); ACC = anterior cingulate cortex; AMY = amygdala; aSLF = anterior superior longitudinal fasciculus; ATC = anterior temporal cortex; CA1 = cornu ammonis 1; CAU = caudate nucleus; GD = gyrus dentatus; IC = internal capsule; IPL = inferior parietal lobule; MFC = middle frontal cortex; pSLF = posterior superior longitudinal fasciculus; SUB = subiculum; TEC = transentorhinal cortex Tau burden (y-axis, a-b) corresponds to the digitally measured %AO by tau pathology after applying natural logarithmic transformation

**Fig. 7:**
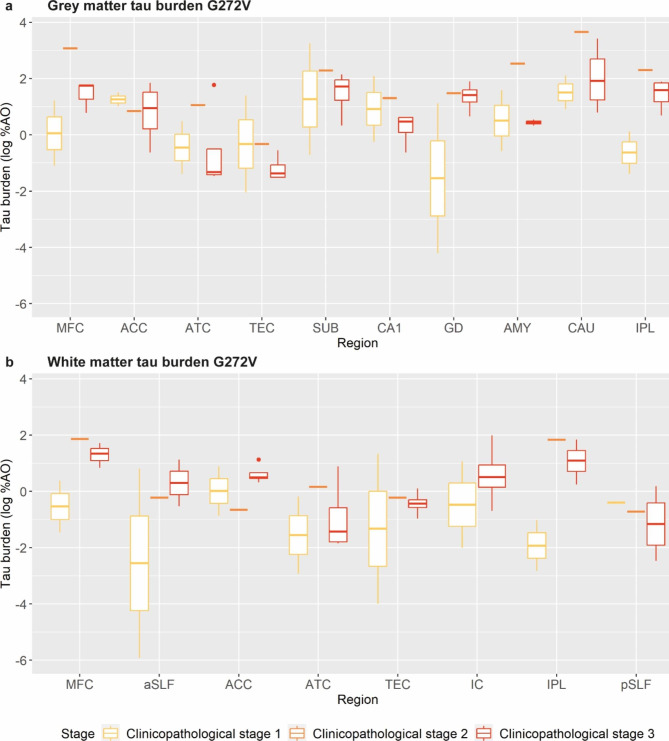
Staging of grey and white matter tau pathology across clinicopathological stages in the G272V variant Plots portray regional progression of grey **(a)** and white **(b)** matter pathology across clinicopathological stages in the G272V variant Legend: %AO = percentage of area occupied (by tau-positive pixels); ACC = anterior cingulate cortex; AMY = amygdala; aSLF = anterior superior longitudinal fasciculus; ATC = anterior temporal cortex; CA1 = cornu ammonis 1; CAU = caudate nucleus; GD = gyrus dentatus; IC = internal capsule; IPL = inferior parietal lobule; MFC = middle frontal cortex; pSLF = posterior superior longitudinal fasciculus; SUB = subiculum; TEC = transentorhinal cortex Tau burden (y-axis, a-b) corresponds to the digitally measured %AO by tau pathology after applying natural logarithmic transformation

Finally, we looked at the progression of tau burden across clinicopathological stages in the P301L (Fig. 6) and G272V variants (Fig. 7), as larger sample sizes were available (≥ 7 cases per variant), distinguishing cases with clinicopathological stage 1 (i.e. FTLD-CDR ≤ 2, 3 P301L, 2 G272V), clinicopathological stage 2 (i.e. FTLD-CDR = 3 & median NDS < 3, 7 P301L, 1 G272V) and clinicopathological stage 3 (i.e. FTLD-CDR = 3 & median NDS ≥ 3, 1 P301L, 4 G272V).

In P301L GM, at clinicopathological stage 1, the frontotemporal cortical regions (ATC, ACC, MFC), the IPL and AMY were affected early. At clinicopathological stage 2, the ACC and ATC showed increasing GM tau burden, while medial temporal regions (TEC, SUB, CA1, GD) also developed comparable high levels of GM tau burden. The MFC and IPL, instead, remained relatively stable in stage 2 compared to stage 1. At clinicopathological stage 3, GM tau burden in most regions decreased, likely due to concomitant increasing neuronal degeneration, except for CAU and IPL that remained stable. In P301L WM, at clinicopathological stage 1, several WM regions were similarly affected early on (MFC, aSLF, ACC, ATC, IC, IPL). At clinicopathological stage 2, a few regions showed decreased WM tau burden (MFC, aSLF, ATC), others stable burden (ACC, IPL), or slightly increased burden (IC, TEC, pSLF). At clinicopathological stage 3, WM tau burden decreased in all regions, likely related to highly degenerated white matter.

In G272V GM, at clinicopathological stage 1, the ACC, SUB, CA1 and CAU were early affected regions, with relatively high tau burden already. At clinicopathological stage 2, GM tau burden increased further in SUB, CA1 and CAU, while it remained stable in ACC. Additional increases in GM tau burden were seen in MFC, ATC, GD and IPL. At clinicopathological stage 3, GM tau burden decreased in most regions (MFC, ATC, TEC, SUB, CA1, CAU, IPL), likely due to increasingly severe neuronal degeneration, or remained relatively stable (ACC, GD). In G272V WM, the ACC, IC and pSLF were early affected regions. At clinicopathological stage 2, other regions reached similar levels of WM tau burden severity (aSLF, ATC, TEC) or even higher (IPL, MFC). Lastly, at clinicopathological stage 3, WM tau burden remained relatively stable in most regions or raised even further (aSFL, ACC, IC).

## Discussion

The current study on the earliest stages of tau pathology in FTLD-MAPT is unique because of the availability of rare brain samples from presymptomatic and early-stage carriers of *MAPT* variants. Additionally, we compared early-stage FTLD-MAPT pathology with intermediate- and end-stage pathology, enabling a clinically driven staging model of pathology in this group, which is a novel approach for FTLD. We found mild-to-moderate tau burden and neuronal degeneration at the early and even presymptomatic stage, yet the morphology and distribution of tau burden differed between the three carriers with distinct *MAPT* variants and associated tau isoforms [26]. The L315R presymptomatic carrier was characterized by pronounced medial temporal tau burden, in part due to concomitant age-related AD neuropathological change. The P301L early-stage carrier showed early involvement of frontotemporal and inferior parietal regions, next to widespread white matter involvement. The G272V early-stage carrier had frontal and temporal (para-)limbic and striatal regions as early-affected grey matter regions. The progression of tau burden through clinicopathological stages differed in part between specific variants. In contrast, neuronal degeneration, mild-to-moderate at the early stage, was distributed quite similarly across variants despite variable levels of tau burden. These data corroborate previous findings pointing to different patterns of tau pathology, but shared network vulnerability to degeneration, between tau isoform groups in FTLD-MAPT [26]. Moreover, through a detailed clinicopathological characterization of this rare cohort, we provide novel insights of early-stage disease features of FTLD-MAPT.

In all three early-stage and presymptomatic carriers, we found tau inclusions morphologically consistent with mature pathological inclusions of each *MAPT* variant described [26], as well as mild-to-moderate neuronal degeneration. These observations suggest that microscopic disease is already present at the early and even presymptomatic stage. To our knowledge, early-stage or presymptomatic pathology in tauopathies has not been described before. In FTLD-TDP with *C9orf72* repeat expansions, presymptomatic pathology (5 years before disease onset) occurs as RNA foci, dipeptide repeat protein inclusions, and loss of nuclear TDP-43, without the TDP-43-positive inclusions found in later stages of the disease [73]. These early pathological features can also be associated with significant degeneration and clinical disease [53, 73], suggesting that mature TDP-43 inclusions are a later phenomenon and not the initial trigger for neuronal damage in this group. As for tau pathology, non-argyrophilic pretangles, as the early stages of intraneuronal tau accumulation in AD [3], precede the formation of beta-amyloid plaques and the occurrence of overt clinical disease by many years [10, 60]. In tauopathies, early morphologies have been described for astrocytic inclusions as well [36], and it has been suggested that changes in astroglial reactivity and functionality may occur early on, preceding neurodegeneration and protein aggregation [35, 42, 47, 48, 57, 58, 65, 71, 76]. Here, we observed the occurrence of (early) astrocytic tau accumulation in the two variants known to have substantial glial burden (P301L, L315R), while neuronal tau accumulation was common to all three variants, consistent with the morphologies of mature pathology. Mild-to-moderate severity of tau burden was associated with young-onset clinical disease in the two early-stage carriers. Such association suggests that tau aggregation in these tauopathies may be more neurotoxic than in AD, where limited tangle formation does not necessarily lead to clinical disease [52]. Gene-related neurotoxic mechanisms may also play a role in the pathogenesis of FTLD-MAPT, making neurons more vulnerable to neurodegeneration, thereby accelerating the disease process [69]. While tau burden was slightly more severe in the upper laminae but extended to the lower laminae as well, neuronal degeneration was often limited to the upper laminae. This observation points to a probable timeline of spatial spread of tau pathology and subsequent degeneration (first upper, then lower laminae), and supports the hypothesis that tau accumulation precedes overt neuronal degeneration in tauopathies [20, 28, 29].

Through this study, we demonstrate for the first time the occurrence of presymptomatic microscopic disease in FTLD-MAPT. These findings are in line with prior antemortem imaging evidence, showing atrophic changes or white matter alterations in the presymptomatic stage of genetic FTD [34, 50, 51, 68]. Additionally, an earlier study using tau PET found subtle increases in binding potential in presymptomatic *MAPT* carriers [74]. However, currently used tracers seem to work well only for specific *MAPT* variants (R406W, V337M) characterized by Alzheimer-like paired helical filaments that contain both 3R and 4R tau isoforms [66, 67, 74]. Importantly, this first report of pathologically proven presymptomatic tau accumulation in FTLD-MAPT reinforces the need and potential value of tau tracers for the presymptomatic prediction of incipient disease. Based on our data, we are not able to assert with certainty how much time lag exists between the formation of inclusions and the occurrence of clinically overt disease. This is an important question, which could be answered through future longitudinal studies following presymptomatic carriers up to conversion with optimized tau PET tracers.

Early-stage patterns of tau burden differed between the three described variants. The presymptomatic carrier with the L315R variant showed limited *MAPT*-related tau burden and mild neuronal degeneration. This variant has been shown to have incomplete penetrance [72], so it is unclear whether this carrier would have ever developed FTD symptoms. This phenomenon may be due to a relatively limited effect of this variant, compared to others, on microtubule assembly [72]. Pathologically, we observed, next to age-related AD neuropathological change in the hippocampal and parahippocampal areas, mild pathological features consistent with the L315R variant, in particular Pick body-like inclusions and astrocytic inclusions (punctate and ramified astrocytes) [26, 72]. Only a few carriers of this variant have been reported in the Netherlands thus far [56, 72], preventing a more extensive staging analysis. Additionally, due to missing tissue from the anterior cingulate cortex, we could not evaluate this brain region, which has been found to be severely affected in variants expressing mixed 3R and 4R tau isoforms such as the L315R [26]. On the other hand, the striatum, a region with high tau burden (but mild-moderate neurodegeneration) in variants expressing mixed 3R and 4R tau isoforms at end-stage, was relatively spared in this presymptomatic carrier, suggesting that this region may become affected at later stages.

The P301L variant showed particular involvement of anterior temporal and frontal regions, and overall of the white matter, at the early stage. These findings align with neuropathological reports of prominent antero-inferior temporal and medial frontal burden observed at end-stage [26], and with the relative abundance of glial inclusions in this variant, such as globular oligodendrocytic inclusions and coiled bodies, and of white matter threads [18, 26]. Further, the early regional pathological involvement reported here, originating in ATC, ACC, MFC, SFC and FIC regions, with relative sparing of medial temporal regions, is consistent with a frontotemporal pattern of atrophy recently observed in this variant through antemortem MRI imaging [77]. Our clinicopathological staging model also supported these early findings, and highlighted the progression to more medial temporal disease at clinicopathological stage 2. At clinicopathological stage 3, a relative decrease in GM tau burden was observed, which is in line with prior findings of plateauing or decreasing tau immunoreactivity at advanced disease stages, due to severe tissue damage and neuronal degeneration [26]. Only one P301L case reached clinicopathological stage 3, while most cases were identified as stage 2, substantiating the idea that 4R tau is less prone to causing very severe degeneration compared to 3R tau [26]. Interestingly, the early-affected IPL region remained stable throughout these stages, similar to observations in sporadic Pick’s disease [31], suggesting that this region may be less vulnerable to tau-induced pathological effects. At severe disease stages, white matter pathology may also undergo substantial degeneration and concomitant loss of tau immunoreactivity, which we observed for this variant at clinicopathological stage 3. This may be due to both Wallerian mechanisms, downstream from affected neurons, and to intrinsic effects of white matter pathological inclusions [25]. These observations emphasize the difficulties of studying end-stage tissue only for the staging of pathological spread in FTLD-MAPT.

The G272V variant, instead, showed early involvement of frontal neocortical and frontotemporal (para-)limbic regions, including the superior frontal cortex, hippocampus, cingulate cortex and striatum. These findings were further supported by patterns of antemortem volume loss in the early-stage G272V case (Suppl. Figure 3), and are consistent with patterns of highest pathological severity observed at end-stage [26]. This variant tended to affect medial temporal regions (such as the subiculum and CA1) early on, differently from the P301L variant. Medial temporal regions showed right lateralization of tau burden, and in part of neuronal degeneration in the subiculum, consistent with findings of right-sided lateralization on MRI (Fig. 3g). Middle frontal lateralization was not evident in postmortem tissue, which may be due to intraregional variability in this large cortical region. The striatum was also prominently involved early on, which is in line with our previous observations of severe striatal tau accumulation in the group of variants with 3R-predominant tau [14, 26]. At the latest clinicopathological stage, severe neuronal degeneration was associated with a prominent decrease in GM tau burden, supporting the hypothesis that 3R tau accumulation may rapidly lead to severe toxic neuronal consequences [26]. On the other hand, early white matter tau pathology was overall less pronounced (compared to the P301L variant), in part due to the rarity of glial inclusions in this variant [14, 26]. Nevertheless, white matter tau burden, primarily occurring as axonal threads, did show a relative increase at higher clinicopathological stages (i.e. 2–3), suggesting that neuronal axons may serve as one possible channel of pathology spread through the white matter.

Neuronal degeneration was distributed similarly when comparing the three early-stage carriers, especially throughout neocortical frontotemporal regions. A similar phenomenon has been observed in our prior study of end-stage FTLD-MAPT, where variants grouped based on the predominant tau isoform showed a partly different distribution of tau burden at end-stage, but similarly showed the most severe neuronal degeneration in the anterior temporal lobe [26]. This suggests that different variants, acting through somewhat different mechanisms [69], cause tau accumulation at distinct regional nodes, but likely affect common frontotemporal (para-)limbic networks, leading to similar atrophy patterns and subsequent functional impairment. This hypothesis is consistent with theories of network-based vulnerability, which have been supported by antemortem imaging studies [61–63, 78]. Interestingly, neuronal degeneration correlated better than tau burden with clinical severity, suggesting that the distribution of neuronal degeneration patterns may reflect clinical symptomatology better than the regional extent of inclusions. This may also explain why these heterogeneous variants (with tau inclusions of different conformation and distribution) end up causing similar clinical disease, a phenomenon which has been referred to as clinicoanatomical convergence [61]. Cellular, laminar or regional vulnerability may also play a role in the degree of involvement of specific regions [19, 41, 49, 63]. For instance, in the P301L group both ATC and IPL showed similar early levels of tau pathology but different subsequent progression; the former became a hotspot for neurodegeneration, while the latter remained relatively spared at later disease stages.

This study presents unique data of early-stage tissue, not reported before for this rare disease. Using thorough digital and semi-quantitative assessments and extensive sampling of available brain tissue, we obtained a rich dataset including a high number of regions relevant for FTLD-MAPT. Some limitations should, however, be considered. The number of these rare early-stage carriers was limited; therefore, these (early) staging patterns should be further verified in other postmortem cohorts, and in more widely available antemortem data with sensitive and pathology-specific antemortem imaging techniques. Our regional selection focused on highly affected regions in FTLD-MAPT; however, other regions that are less or variably affected in *MAPT* variants (e.g. brain stem nuclei) should be investigated in follow-up studies. Since *MAPT* variants show significant heterogeneity, we attempted to perform sub-analyses within each variant group; yet, power for statistical analyses was limited, and some of the observations were only qualitative. Additionally, we had a small amount of missing data due to unavailable tissue for these legacy autopsy cases. As tissue was harvested from one hemisphere only for most cases, we cannot exclude the potential influence of hemispheric differences, although neuroimaging studies of cortical atrophy suggest that FTLD-MAPT progresses quite symmetrically across hemispheres [16, 55]. Lateralization may be more evident in the early stage, as observed in the G272V early-stage carrier, who was primarily sampled in the left hemisphere but showed right-lateralized tau pathology. In the L315R presymptomatic carrier, age-related co-pathology limited in part the assessment of tau pathology uniquely related to the L315R variant. Tau pathology assessment was based on anti-AT8 immunohistochemistry, which is a well-established marker for early and mature tau pathology [6, 9, 45]; however, stage-specific tau markers, immunoblotting and advanced imaging techniques (e.g. cryo-electron microscopy [59, 64]) could help to further characterize the structural and biochemical properties of pathological inclusions in early-stage FTLD-MAPT [45]. Finally, we focused on tau distribution and neurodegeneration in this study, yet other markers (e.g. astrogliosis [13, 35, 65], microgliosis [13, 39, 75], neurovascular markers [21]) could provide important insights into related disease dynamics, and help to figure out the chronology of neuroinflammatory changes in this unique early-stage tissue.

To conclude, we describe a set of three unique presymptomatic/early-stage *MAPT* variant carriers, showing early pathological features of tau burden, with somewhat distinct distribution patterns between these variants, and quite similar frontotemporal (para-)limbic neuronal degeneration. These findings help to understand the pathophysiology of *MAPT-*associated disease, and highlight the potential role of upcoming tau-specific markers for the early, and possibly presymptomatic, detection of incipient disease.

## Electronic supplementary material

Below is the link to the electronic supplementary material.

Supplementary Material 1: Online supplement: Supplementary Tables 1-2; Supplementary Figures 1-5

## Data Availability

Data from this study will be made available by the corresponding authors upon reasonable request.
