# Supplementary material for: Presymptomatic and early pathological features of MAPT-associated frontotemporal lobar degeneration
Source: Acta Neuropathol Commun. 2023 Aug 2;11:126. doi: 10.1186/s40478-023-01588-9 (PMC10394953; doi:10.1186/s40478-023-01588-9)

**Online supplement for:**

**Presymptomatic and early pathological features of
*MAPT*-associated frontotemporal lobar degeneration**

Lucia AA Giannini^1^, Merel O Mol^1^, Ana Rajicic^1^, Renee van Buuren^1^, Lana Sarkar^1^,
Sanaz Arezoumandan^2,3^, Daniel T Ohm^2,3^, David J Irwin^2,3^, Annemieke JM Rozemuller^4^,
Netherlands Brain Bank^5^, John C van Swieten^1^, Harro Seelaar^1^

**Author affiliations:**

^1^Department of Neurology and Alzheimer Center Erasmus MC, Erasmus University Medical Center, 3015 GD, Rotterdam, the Netherlands

^2^Digital Neuropathology Laboratory, Department of Neurology, Perelman School of Medicine, University of Pennsylvania, PA 19104, Philadelphia, USA

^3^Penn Frontotemporal Degeneration Center, Department of Neurology, Perelman School of Medicine, University of Pennsylvania, PA 19104, Philadelphia, USA

^4^Department of Pathology, Amsterdam Neuroscience, Amsterdam University Medical Center, location VUmc, 1081 HZ, Amsterdam, The Netherlands

^5^Netherlands Institute for Neuroscience, Meibergdreef 47, 1105 BA, Amsterdam

Correspondence to:

Harro Seelaar, MD, PhD

Alzheimer Center, Department of Neurology

Erasmus University Medical Center

Doctor Molewaterplein 40

3015 GD Rotterdam

[h.seelaar@erasmusmc.nl](mailto:h.seelaar@erasmusmc.nl)

2 Supplementary Tables, 5 Supplementary Figures

**Supplementary Table 1. Overview of available %AO measurements per region**

| **Cortical grey matter** | **MFC** | **SFC** | **ACC** | **FIC** | **ATC** | **FG** | **TEC** | **EC** | **IPL** | **Total** |
| --- | --- | --- | --- | --- | --- | --- | --- | --- | --- | --- |
| Presympt L315R (n = 1) | 1 | 0 | 0 | 0 | 1 | 1 | 1 | 1 | 1 | 6 |
| Intermed/Late L315R (n = 2) | 2 | 1 | 1 | 2 | 2 | 2 | 2 | 2 | 2 | 16 |
| Early G272V (n = 1) | 2 | 1 | 1 | 0 | 1 | 2 | 2 | 2 | 1 | 12 |
| Intermed/Late G272V (n = 6) | 5 | 6 | 6 | 6 | 6 | 6 | 6 | 6 | 6 | 53 |
| Early-stage P301L (n = 1) | 1 | 1 | 1 | 1 | 1 | 0 | 1 | 1 | 1 | 8 |
| Intermed/Late P301L (n = 10) | 9 | 8 | 9 | 7 | 10 | 7 | 9 | 9 | 10 | 78 |
| Total | 20 | 17 | 18 | 16 | 21 | 18 | 21 | 21 | 21 | 173 |
| **Subcortical grey matter** | **SUB** | **CA1** | **CA2** | **CA3** | **CA4** | **GD** | **AMY** | **PUT** | **CAU** | **Total** |
| Presympt L315R (n = 1) | 1 | 1 | 1 | 1 | 1 | 1 | 1 | 1 | 1 | 9 |
| Intermed/Late L315R (n = 2) | 2 | 2 | 1 | 1 | 1 | 1 | 2 | 2 | 2 | 14 |
| Early G272V (n = 1) | 2 | 2 | 2 | 2 | 2 | 2 | 1 | 1 | 1 | 15 |
| Intermed/Late G272V (n = 6) | 6 | 6 | 6 | 6 | 6 | 6 | 4 | 4 | 6 | 50 |
| Early-stage P301L (n = 1) | 1 | 1 | 1 | 1 | 1 | 1 | 1 | 1 | 1 | 9 |
| Intermed/Late P301L (n = 10) | 10 | 10 | 10 | 10 | 10 | 10 | 10 | 7 | 7 | 84 |
| Total | 22 | 22 | 21 | 21 | 21 | 21 | 19 | 16 | 18 | 181 |
| **Juxtacortical white matter** | **MFC** | **SFC** | **ACC** | **FIC** | **ATC** | **FG** | **TEC** | **EC** | **IPL** | **Total** |
| Presympt L315R (n = 1) | 1 | 0 | 0 | 0 | 1 | 1 | 1 | 1 | 1 | 6 |
| Intermed/Late L315R (n = 2) | 1 | 1 | 1 | 2 | 2 | 2 | 2 | 2 | 2 | 15 |
| Early G272V (n = 1) | 2 | 1 | 1 | 0 | 1 | 2 | 2 | 2 | 1 | 12 |
| Intermed/Late G272V (n = 6) | 5 | 6 | 6 | 6 | 6 | 6 | 6 | 6 | 6 | 53 |
| Early-stage P301L (n = 1) | 1 | 1 | 1 | 1 | 1 | 0 | 1 | 1 | 1 | 8 |
| Intermed/Late P301L (n = 10) | 9 | 8 | 9 | 7 | 9 | 7 | 9 | 9 | 10 | 77 |
| Total | 19 | 17 | 18 | 16 | 20 | 18 | 21 | 21 | 21 | 171 |
| **Subcortical white matter** | **aSLF** | **CC** | **IC** | **pSLF** |  |  |  |  |  | **Total** |
| Presympt L315R (n = 1) | 1 | 0 | 1 | 1 |  |  |  |  |  | 3 |
| Intermed/Late L315R (n = 2) | 2 | 1 | 2 | 2 |  |  |  |  |  | 7 |
| Early G272V (n = 1) | 2 | 0 | 1 | 0 |  |  |  |  |  | 3 |
| Intermed/Late G272V (n = 6) | 4 | 5 | 5 | 6 |  |  |  |  |  | 20 |
| Early-stage P301L (n = 1) | 1 | 1 | 1 | 1 |  |  |  |  |  | 4 |
| Intermed/Late P301L (n = 10) | 7 | 8 | 7 | 9 |  |  |  |  |  | 31 |
| Total | 17 | 15 | 17 | 19 |  |  |  |  |  | 68 |

Legend: ACC = anterior cingulate cortex; AMY = amygdala; aSLF = anterior superior longitudinal fasciculus; ATC = anterior temporal cortex; CA1-4 = cornu ammonis 1-4; CAU = caudate nucleus; CC = corpus callosum; EC = entorhinal cortex; FIC = fronto-insular cortex; FG = fusiform gyrus; GD = gyrus dentatus; IC = internal capsule; Intermed = intermediate; IPL = inferior parietal lobule; MFC = middle frontal cortex; Presympt = presymptomatic; pSLF = posterior superior longitudinal fasciculus; PUT = putamen; SFC = superior frontal cortex; SUB = subiculum; TEC = transentorhinal cortex.

**Supplementary Table 2. Correlation of tau burden and neuronal degeneration with clinical severity**

| **GM tau burden** | **rho** | **p-value** | **corrected p-value** |
| --- | --- | --- | --- |
| ACC | 0.15 | 0.56 |  |
| MFC | 0.51 | 0.03 | 0.251 |
| ATC | 0.26 | 0.25 |  |
| TEC | 0.26 | 0.27 |  |
| IPL | 0.54 | 0.01 | 0.115 |
| SUB | 0.33 | 0.14 |  |
| CA1 | 0.24 | 0.29 |  |
| GD | 0.57 | 0.01 | 0.092 |
| AMY | 0.48 | 0.04 | 0.358 |
| CAU | 0.51 | 0.03 | 0.322 |
| **Neuronal degeneration score** | **rho** | **p-value** | **corrected p-value** |
| ACC | 0.67 | 0.00 | 0.025 |
| MFC | 0.73 | 0.00 | 0.004 |
| ATC | 0.63 | 0.00 | 0.021 |
| TEC | 0.69 | 0.00 | 0.008 |
| IPL | 0.73 | 0.00 | 0.002 |
| SUB | 0.55 | 0.01 | 0.100 |
| CA1 | 0.63 | 0.00 | 0.023 |
| GD | 0.62 | 0.00 | 0.039 |
| AMY | 0.48 | 0.04 | 0.384 |
| CAU | 0.77 | 0.00 | 0.002 |
| **WM tau burden** | **rho** | **p-value** | **corrected p-value** |
| ACC | 0.07 | 0.77 |  |
| MFC | 0.18 | 0.47 |  |
| ATC | 0.16 | 0.51 |  |
| TEC | 0.27 | 0.24 |  |
| IPL | 0.46 | 0.04 | 0.281 |
| aSLF | 0.03 | 0.92 |  |
| pSLF | 0.18 | 0.45 |  |
| IC | 0.51 | 0.04 | 0.298 |

Legend: GM = grey matter; WM = white matter.

**Supplementary Figure 1. Sampling of hippocampus based on neuroanatomical and cytoarchitectural features**


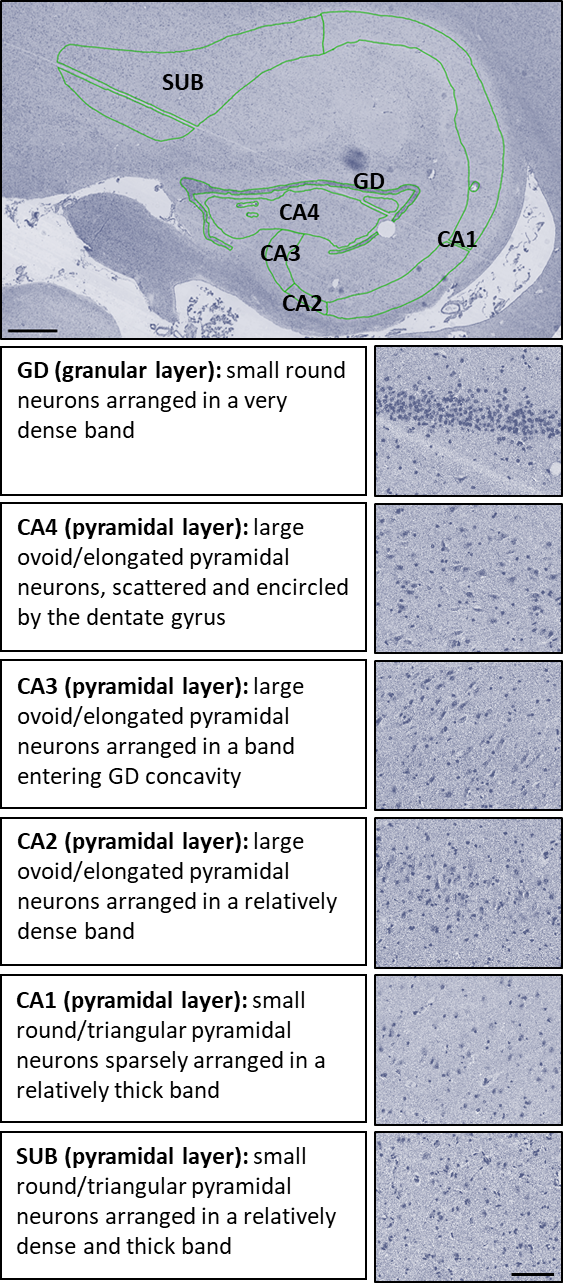

Legend: CA1-4 = cornu ammonis 1-4; GD = gyrus dentatus; SUB = subiculum. Hippocampal subfields were identified and annotated based on the characteristics displayed in figure. Scale = 1000 µm (overview); 100 µm (zoom).

**Supplementary Figure 2. Validation of %AO scores by comparison to traditional ordinal ratings (0-3)**

Legend: %AO = percentage of area occupied (by tau-positive pixels). Quantitative %AO scores (with natural logarithmic transformation) of tau pathology were validated by comparison to conventional ordinal scores (i.e. 0-3). In both grey (a) and white (b) matter; quantitative %AO scores were associated with ordinal scores of tau pathology (p < 0.001); with significant post-hoc differences in %AO between each ordinal score level (p < 0.001).


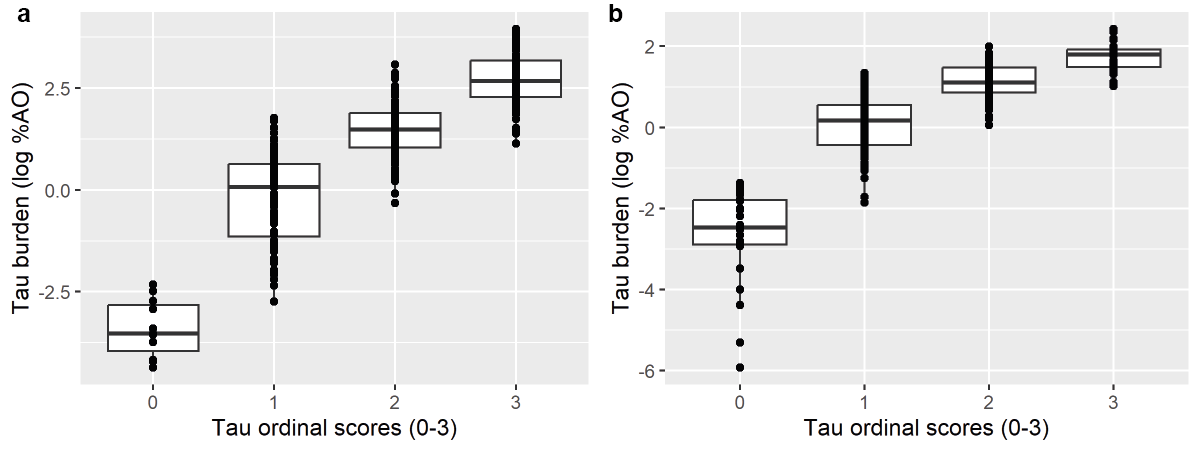


**Supplementary Figure 3. Antemortem MRI volume w-scores in the early-stage G272V carrier**

Plot portrays regional MRI volume loss of the G272V early-stage carrier at the last scan before autopsy (MRI-autopsy interval 0.1 years). MRI volumes were obtained for cortical and subcortical structures matching the postmortem sampling (Hammers atlas in CAT12.8.1, v1975, <http://www.neuro.uni-jena.de/cat/>), corrected for total intracranial volume and W-score-transformed based on an independent cohort of healthy controls (32 males, 47 females, mean age 48.7±12.0 years). Legend: ACC = anterior cingulate cortex; AMY = amygdala; ATC = anterior temporal cortex; CAU = caudate nucleus; FG = fusiform gyrus; FIC = frontoinsular cortex; HIPP = hippocampus; IPL = inferior parietal lobule; MFC = middle frontal cortex; PUT = putamen; SFC = superior frontal cortex; TEC/EC = transentorhinal/entorhinal cortex.


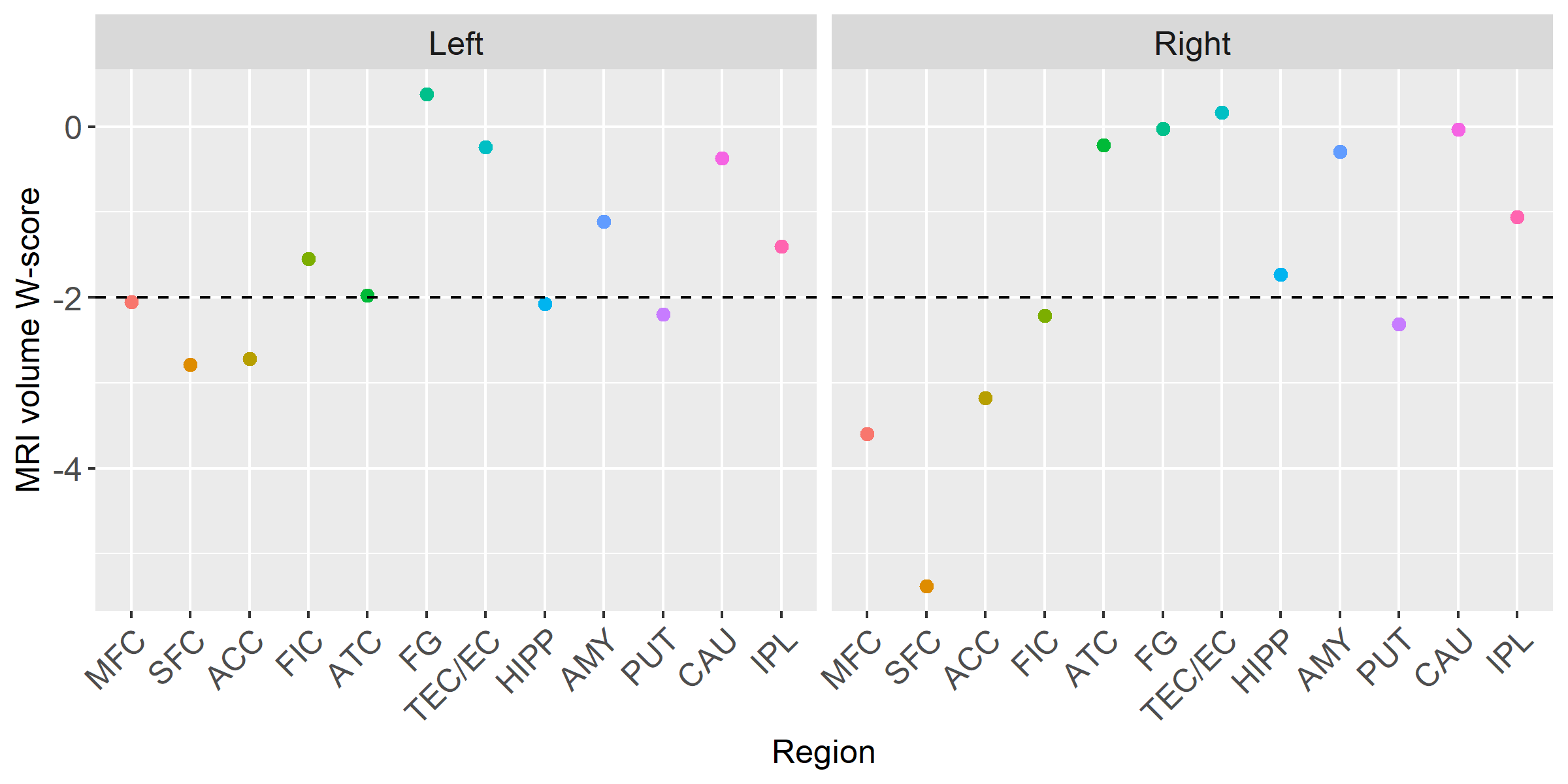


**Supplementary Figure 4. Comparisons of tau burden across clinical stages in each *MAPT* variant**

Legend: %AO = percentage of area occupied (by tau-positive pixels). Plots show the relative levels of tau burden in cortical/subcortical grey matter; and in juxtacortical/subcortical white matter; progressing through clinical stages for each *MAPT* variant.


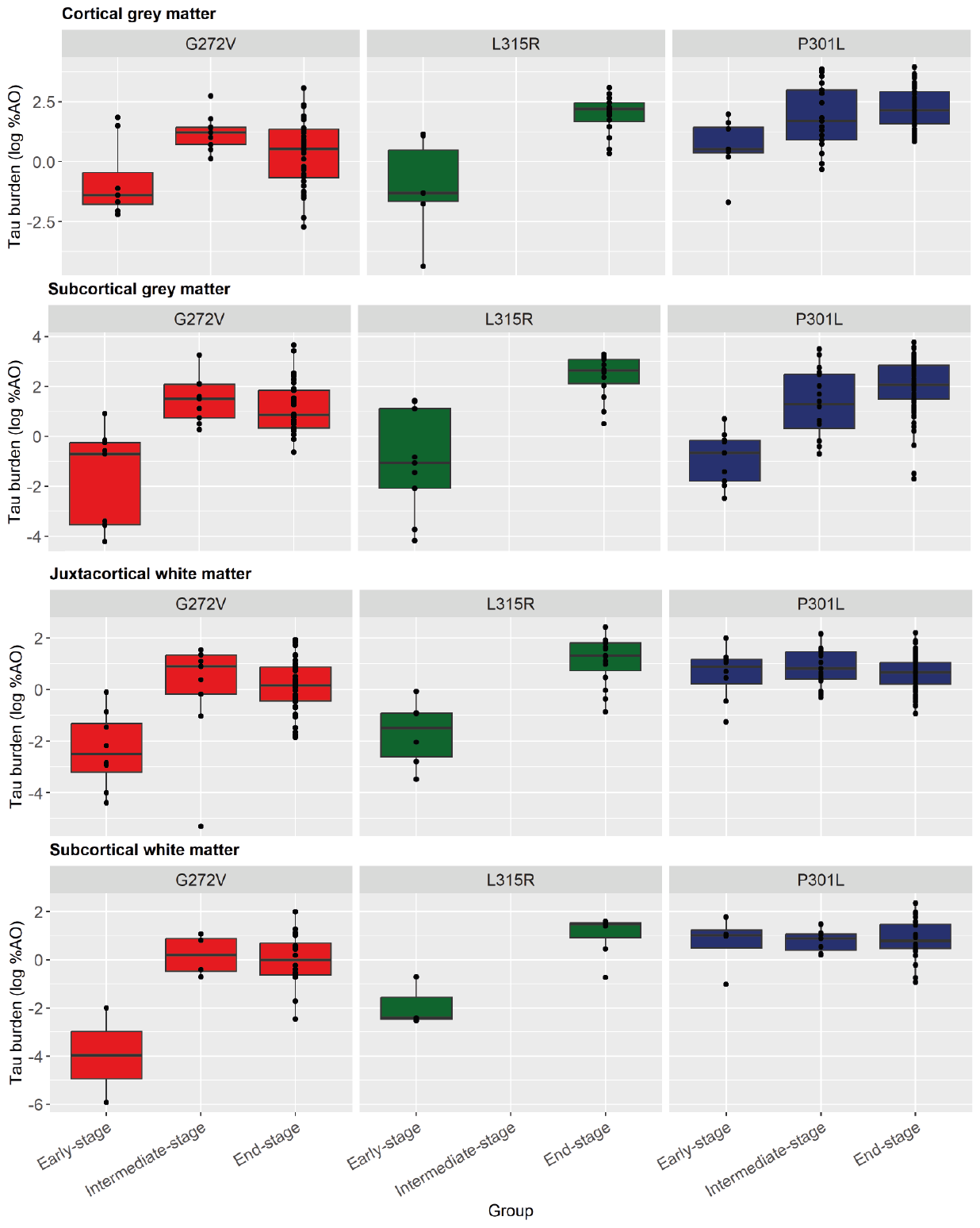


**Supplementary Figure 5. Comparisons of neuronal degeneration across clinical stages in each *MAPT* variant**

Plots show the relative severity of neuronal degeneration in cortical/subcortical grey matter progressing through clinical stages for each *MAPT* variant.


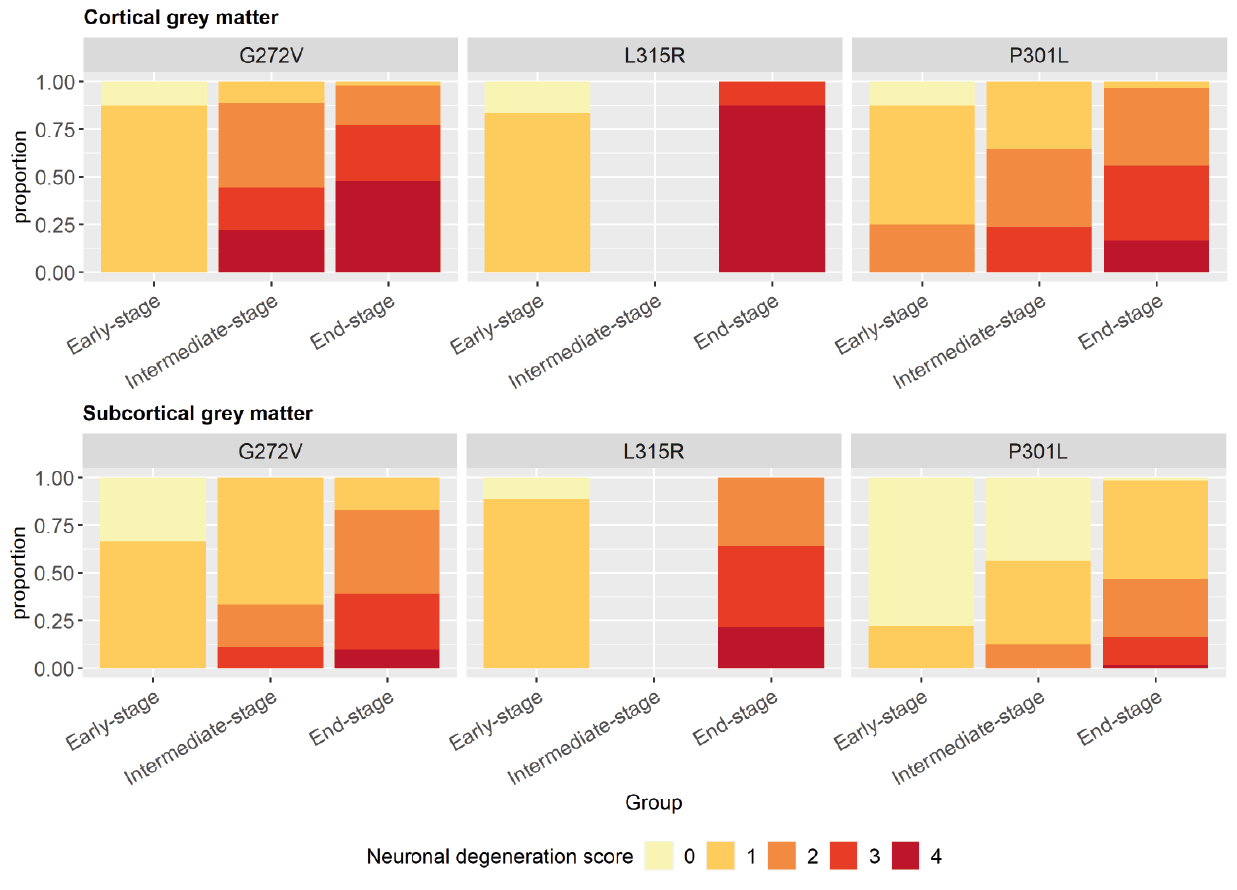

Supplement: Supplementary file 1 — Supplementary Material 1: Online supplement: Supplementary Tables 1-2; Supplementary Figures 1-5 [file 40478_2023_1588_MOESM1_ESM.docx]
